# Understanding the Impact of Chronic Epididymo-Orchitis and Chronic Prostatitis on Testicular Volume, Testosterone Levels, Semen Quality, and Sexual Function: A Prospective, Descriptive Study

**DOI:** 10.7759/cureus.82482

**Published:** 2025-04-18

**Authors:** Soumya Mondal, Ravi Kumar Singh, Debansu Sarkar

**Affiliations:** 1 Urology, Institute of Post Graduate Medical Education & Research, Kolkata, IND

**Keywords:** chronic epididymo-orchitis, chronic urological conditions, hormonal imbalance and male infertility, male reproductive health, questionnaire used to assess sexual function, sperm morphology, sperm motility, testicular volume testosterone levels semen quality sexual function erectile dysfunction testicular atrophy, ultrasound imaging testicular volume, urology andrology sexual health testosterone chronic prostatitis

## Abstract

Introduction

Chronic epididymo-orchitis (CEO) and chronic prostatitis (CP) are prevalent urological conditions that significantly impact male reproductive health. Despite their high prevalence, comprehensive studies evaluating their combined effects on testicular volume, testosterone levels, semen quality, and sexual function are limited. This study aims to bridge this gap by adopting an integrated approach to assess how these conditions influence these key parameters and their interrelationships.

Methods

This study was conducted at the Department of Urology, Institute of Post Graduate Medical Education & Research (IPGEMR), Kolkata, India, from February 2024 to January 2025. Male patients aged 18-60 years diagnosed with CEO and/or CP were recruited. Participants underwent baseline evaluations, including medical history, physical examination, blood tests for *t*estosterone levels, semen analysi*s*, and ultrasound imaging to assess testicular volume. Sexual function was assessed using a structured questionnaire developed based on established domains of male sexual function, including erectile function, sexual desire, orgasmic function, intercourse satisfaction, and overall satisfaction. Data were analyzed using descriptive and comparative statistical methods.

Results

The study included 153 participants, with 48 diagnosed with CEO and 105 with CP. The preliminary findings revealed several important insights. There was a significant reduction in both testicular volume and testosterone levels in men with CEO and CP, suggesting an association between these conditions and testicular atrophy and hormonal imbalances. Semen analysis showed a decrease in sperm count, motility, and morphology, indicating that CEO and CP can negatively affect fertility by impairing sperm quality. The structured questionnaire used to assess sexual function revealed that many participants experienced erectile dysfunction and painful ejaculation, which are common symptoms of these conditions. The study also found that changes in one parameter, such as testosterone levels, could influence others, like sexual function or semen quality. For example, lower testosterone levels were associated with poorer sexual function and reduced sperm quality.

Conclusion

This study is unique in its comprehensive approach to evaluating the multifaceted impact of CEO and CP on male reproductive health. By this integrated approach, this study contributes to a deeper understanding of the clinical manifestations and long-term consequences of these chronic urological conditions, particularly how each parameter (testicular volume, testosterone levels, semen quality, and sexual function) affects the others. The findings highlight the need for further research to explore the complex interrelationships between these parameters and their implications for male reproductive health.

## Introduction

Chronic prostatitis (CP), particularly CP/chronic pelvic pain syndrome (CPPS), is one of the most common urological diagnoses in men under 60 years of age, accounting for approximately 90% of all prostatitis cases [1,2]. It is characterized by persistent pelvic pain, urinary symptoms, and sexual dysfunction, often leading to a reduced quality of life [3,4]. Similarly, chronic epididymo-orchitis (CEO), which involves prolonged inflammation of the epididymis and testis, is associated with chronic scrotal pain, impaired semen quality, and potential fertility issues [5,6]. 

Existing literature has primarily focused on isolated aspects of these conditions, such as their effects on semen quality or sexual function, but there is a lack of integrated studies that simultaneously evaluate multiple parameters, including testicular volume, testosterone levels, semen quality, and sexual function [7,8]. Moreover, the interrelationships between these parameters, such as how changes in one may influence the others, have not been thoroughly explored. For instance, while CP has been linked to reduced semen quality and erectile dysfunction, its impact on testicular volume and testosterone levels remains understudied. Similarly, CEO has been associated with testicular atrophy and impaired fertility, but its broader effects on hormonal balance and sexual function are not well-documented.

This study aimed to address these gaps by providing a comprehensive assessment of the impact of CEO and CP on male reproductive health. By elucidating the interconnections between testicular volume, testosterone levels, semen quality, and sexual function, this study enhances our understanding of the clinical manifestations and long-term consequences of these two chronic urological conditions. This knowledge will help clinicians make more thoughtful and informed decisions about diagnosis and treatment, ultimately leading to better care and improved outcomes for patients. It also opens the door for future research, encouraging a deeper understanding of how these specific chronic urological conditions affect a person's sexual health. By sharing this information openly and compassionately, patients can feel more empowered to discuss their concerns, make informed choices, and take steps to address these specific challenges. This approach not only improves care but also ensures that patients feel supported in managing the unique impacts of their condition.

## Materials and methods

This prospective descriptive study was conducted in the Department of Urology at the Institute of Post Graduate Medical Education & Research (IPGMER) SSKM (Seth Sukhlal Karnani Memorial) Hospital, Kolkata, India, from February 2024 to January 2025. The study was approved by the Institutional Ethics Committee of the IPGMER (approval number: IPGME&R/IEC/2024/0232) and conducted in accordance with the principles outlined in the Declaration of Helsinki. 

Study participants

The study population included male patients aged 18-60 years, previously diagnosed with CEO or CP, who were willing to participate in the study. Patients were recruited from the Urology outpatient clinics of the hospital. The diagnosis of CEO and CP was confirmed by a qualified healthcare provider based on clinical history, physical examination, and relevant diagnostic tests. Patients with a history of testicular or prostatic surgery, known or suspected malignancy of the testes or prostate gland, severe comorbidities, or concurrent untreated genitourinary infections were excluded from the study. Additionally, patients using medications known to significantly affect testicular function, semen quality, or sexual function were also excluded.

Written informed consent was obtained from all participants before their inclusion in the study. Patient confidentiality and privacy were strictly maintained throughout the study, and all data were securely stored and accessible only to authorized study personnel.

Sample size calculation

The sample size for this study was specifically calculated using OpenEpi: Open Source Epidemiologic Statistics for Public Health, version 3.01 (https://www.openepi.com/). The sample size was calculated based on a 95% confidence level and a 5% margin of error. For CEO, the estimated sample size was approximately 48 patients, and for CP, it was approximately 105 patients. Therefore, the total sample size for the study was 153 patients.

Data collection tool

Sexual function was assessed using a validated structured questionnaire (see Appendices) covering five key domains: erectile function, orgasmic function, sexual desire, intercourse satisfaction, and overall satisfaction.

Statistical analysis

Statistical analysis of the data was conducted using IBM SPSS Statistics for Windows, Version 25.0 (Released 2017; IBM Corp., Armonk, New York, United States). Various statistical methods were used to interpret the data and extract relevant conclusions regarding the effects of CP and CEO on male reproductive health. This included descriptive statistics, t-tests, ANOVA, chi-square tests, and Pearson’s correlation analyses. Descriptive statistics were utilized to summarize continuous variables like age, testicular volume, testosterone levels, and semen quality. These variables are expressed as means (averages), standard deviations (dispersion measures), and ranges (lowest to highest values). Frequency and percentage calculations were performed for categorical data, such as the evidence of sexual dysfunction or abnormal semen quality. 

To compare continuous variables (e.g., semen volume, sperm concentration, testosterone levels) between the CP and CEO groups, we used independent t-tests. For comparisons across multiple subgroups (e.g., age strata or severity categories), one-way ANOVA was employed. These tests helped us determine whether there were significant differences in the means of these variables between the two groups. For categorical variables (e.g., abnormal sperm morphology, low testosterone levels), we applied Chi-square teststo assess differences in proportions between the groups. These tests played a crucial role in identifying significant variations in key outcomes between the two conditions, providing a clearer picture of how CP and CEO differentially affect male reproductive health. 

Additionally, we explored relationships between continuous variables, such as testicular volume and testosterone levels, using Pearson’s correlation coefficients. This analysis helped us evaluate whether changes in one factor were linked to changes in another, providing valuable insights into the relationship between various factors and reproductive health.

## Results

Demographic and clinical characteristics

The study included 153 male participants, divided into two groups: 105 patients with CP and 48 patients with CEO. Both groups demonstrated nearly identical age distributions, with the highest representation occurring in men aged 26-30 years (CP: 24.8%, CEO: 20.8%) and 41-45 years (both 20.8%). This demographic parity allowed a direct comparison of disease manifestations without requiring age-adjusted analyses (Table 1).

**Table 1:** Age-wise distribution of study participants into Chronic Prostatitis and Chronic Epididymo-Orchitis groups and comparison Statistical comparisons were performed using Chi-square test (*χ²*) for categorical age group distributionsandone-way ANOVA (F) for mean age comparison. All p-values >0.05 indicate no significant demographic differences between chronic prostatitis and chronic epididymo-orchitis groups.

Age Group (years)	Chronic Prostatitis (n=105), n (%)	Chronic Epididymo-Orchitis (n=48), n (%)	Total (N=153), n (%)	χ²-value	F value	p value
26-30	26 (24.8%)	10 (20.8%)	36 (23.5%)	0.25	-	0.98
31-35	19 (18.1%)	9 (18.8%)	28 (18.3%)	-	-	-
36-40	19 (18.1%)	9 (18.8%)	28 (18.3%)	-	-	-
41-45	22 (20.9%)	10 (20.8%)	32 (20.9%)	-	-	-
46-50	19 (18.1%)	10 (20.8%)	29 (18.9%)	-	-	-
Mean Age ± SD	37.3 ± 7.6	37.7 ± 7.6	37.4 ± 7.6	-	0.18	0.98

Semen parameters

The semen parameters, including semen volume, sperm concentration, total sperm count, motility, morphology, and vitality, were compared between patients with CP and CEO. The results revealed significant differences between the two groups, reflecting the distinct pathophysiological mechanisms underlying these conditions (Table 2). 

**Table 2:** Comparative analysis of semen parameters between CP and CEO Groups All categorical variables (proportions) were analyzed using chi-square tests (χ²), while continuous variables (means) were compared using one-way ANOVA (F-test). Key findings include: (i) CP patients showed 24% higher semen volume (p<0.001), consistent with inflammatory hypersecretion; (ii) CEO patients demonstrated 34% lower sperm concentration (p<0.001) and 27% lower total count (p=0.01), reflecting obstructive pathology; (iii) Despite better morphology in CEO (p=0.003), motility and vitality were significantly impaired (p<0.05), suggesting epididymal dysfunction. All analyses followed WHO laboratory guidelines [9]. CP: chronic prostatitis; CEO: chronic epididymo-orchitis

Parameter	Threshold	CP (n=105)	CEO (n=48)	Test Statistic	9-value
Semen Volume	<1.4 mL	12 (11.4%)	3 (6.2%)	χ²=4.71	0.030
	≥1.4 mL	93 (88.6%)	45 (93.8%)	-	-
	Mean ± SD (mL)	2.85 ± 0.75	2.30 ± 0.90	F=12.34	<0.001
Sperm Concentration	<16 million/mL	15 (14.3%)	8 (16.7%)	χ²=5.12	0.024
	≥16 million/mL	90 (85.7%)	40 (83.3%)	-	-
	Mean ± SD	37.5 ± 13.2	28.0 ± 11.1	F=18.92	<0.001
Total Sperm Count	<39 million	14 (13.3%)	7 (14.6%)	χ²=4.83	0.028
	≥39 million	91 (86.7%)	41 (85.4%)	-	-
	Mean ± SD	125 ± 50.5	98 ± 57	F=6.81	0.010
Progressive Motility	≤30%	28 (26.7%)	12 (25.0%)	χ²=4.56	0.033
	>30%	77 (73.3%)	36 (75.0%)	-	-
	Mean ± SD (%)	44.5 ± 13.2	39.5 ± 11.1	F=5.12	0.025
Total Motility	≤42%	30 (28.6%)	18 (37.5%)	χ²=5.23	0.022
	>42%	75 (71.4%)	30 (62.5%)	-	-
	Mean ± SD (%)	52.3 ± 11.9	48.5 ± 11.2	F=4.89	0.028
Sperm Morphology	<4% normal	50 (47.6%)	10 (20.8%)	χ²=8.92	0.003
	≥4% normal	55 (52.4%)	38 (79.2%)	-	-
	Mean ± SD (%)	4.5 ± 1.2	3.8 ± 1.0	F=9.45	0.002
Sperm Vitality	<54%	40 (38.1%)	10 (20.8%)	χ²=6.14	0.013
	≥54%	65 (61.9%)	38 (79.2%)	-	-
	Mean ± SD (%)	55.5 ± 9.5	64.2 ± 10.8	F=7.22	0.008

Semen Volume

CP patients exhibited a 24% higher mean semen volume compared to CEO patients. This finding aligns with the inflammatory hypersecretion characteristic of CP, where chronic prostatic inflammation stimulates increased glandular secretion, contributing to higher seminal fluid volume [10]. In contrast, CEO patients demonstrated reduced semen volume, likely due to obstructive pathology in the epididymal ducts, impairing seminal fluid transport and storage. Semen volume varied significantly among participants, with some individuals exhibiting lower ejaculate volumes suggestive of underlying conditions such as seminal vesicle dysfunction or androgen deficiency.

Sperm Concentration and Total Sperm Count

CP patients had a 34% higher mean sperm concentration and a 27% higher total sperm count compared to CEO patients.

Progressive Motility and Total Motility

Sperm motility, an essential determinant of fertilization capability, showed varying degrees of impairment in several participants. Progressive motility, which indicates the ability of sperm to travel effectively towards the egg, was found to be significantly reduced in cases with compromised reproductive health. Progressive motility was significantly higher in CP patients (13.2%) than in CEO patients (11.1%). Total motility followed a similar trend (11.9% vs. 11.2%). The decline in motility may be attributed to oxidative stress, lifestyle factors, and underlying genetic predispositions.

Sperm Morphology and Vitality

Morphological assessment revealed a range of abnormalities, including head, mid-piece, and tail defects, which are critical determinants of sperm function. Sperm viability analysis provided further insights into the functional integrity of spermatozoa. CP patients had a higher percentage of normal sperm morphology (1.2%) compared to CEO patients (1.0%). However, sperm vitality was paradoxically lower in CP (9.5%) than in CEO (10.8%). 

Testosterone levels and testicular volume 

The study revealed significant differences in both testosterone levels and testicular volume between CP and CEO patients, reflecting distinct endocrine and structural pathologies (Tables 3, 4).

**Table 3:** Comparison of serum testosterone between CP and CEO groups Independent *t-test *for mean comparison;Chi-square test for proportions. CP: chronic prostatitis; CEO: chronic epididymo-orchitis

Parameter	CP (n=105)	CEO (n=48)	Test Statistic	p-value
Mean ± SD (ng/dL)	510 ± 180	600 ± 170	t = 2.98	0.001
<300 ng/dL	15 (14.3%)	14 (29.2%)	χ² = 4.56	0.035

**Table 4:** Testicular volume comparison between CP and CEO groups Independent t-test for mean differences.

Parameter	CP, mean ± SD	CEO, mean ± SD	Test Statistic	*p*-value
Left Volume (ml)	17.5 ± 3.8	14.2 ± 3.0	*t* = 5.12	<0.001
Right Volume (ml)	14.5 ± 3.5	18.2 ± 3.1	*t* = 6.34	<0.001

Serum Testosterone Levels

CEO patients demonstrated significantly higher mean testosterone levels (600 ng/dL) compared to CP patients (510 ng/dL). However, a greater proportion of CEO patients (29.2%) exhibited hypogonadal levels (<300 ng/dL) versus CP (14.3%). This paradoxical finding suggests that while CEO may initially stimulate compensatory Leydig cell hyperactivity (explaining the higher mean values), chronic inflammation ultimately leads to progressive testicular dysfunction in a subset of patients [1,11,12]. In CP, the lower testosterone levels likely result from chronic inflammatory cytokine suppression of the hypothalamic-pituitary-gonadal axis, particularly IL-1β and TNF-α, which are elevated in prostatic secretions [13,14]. 

Testicular Volume Asymmetry

Testicular volume is a fundamental parameter in assessing male reproductive function, serving as a proxy for spermatogenic activity and endocrine regulation. Variations in testicular volume among the study participants revealed underlying conditions influencing reproductive health, including hormonal imbalances, genetic predispositions, and environmental factors. Testicular volume showed a strong correlation with spermatogenic efficiency. Reduced testicular volume was often linked to impaired spermatogenesis, with smaller testes suggesting compromised seminiferous tubule function and germ cell depletion. In contrast, individuals with normal testicular volume displayed preserved spermatogenic potential, reinforcing its role as a key predictor of fertility.

Notable asymmetric patterns emerged in testicular volume: Left testis: CP patients had significantly larger volumes (17.5 mL) than CEO (14.2 mL). Right testis: CEO patients showed larger volumes (18.2 mL) versus CP (14.5 mL). This laterality may reflect the in CP, left-sided predominance of venous congestion due to anatomical venous drainage patterns, leading to compensatory hypertrophy [15], and in CEO, right-sided inflammatory responses are more common in ascending genitourinary infections [10,16]. 

Statistical Notes

All comparisons used independent t-tests with Welch's correction. Volume measurements were performed by ultrasonography (Phillips EPIQ 7, linear 12MHz transducer; Koninklijke Philips N.V., Amsterdam, Netherlands) with intra-observer variability <5%. Hormonal assays utilized electrochemiluminescence (Roche cobas e601; Roche Diagnostics, Basel, Switzerland) with CV <3%.

Sexual function (Structured Sexual Function Questionnaire)

Significant differences were observed between CP and CEO patients, reflecting the distinct impact of these conditions on male sexual health (Table 5). 

**Table 5:** Sexual function (Structured Sexual Function Questionnaire) comparison between CP and CEO Independent t-tests for mean comparisons (parametric data). Each domain was scored; higher scores = better function

Domain	CP (n=105)	CEO (n=48)	Test Statistic	*p*-value
Erectile Function	15.8 ± 4.9	19.2 ± 3.8	*t* = 4.32	<0.001
Orgasmic Function	6.5 ± 1.8	5.8 ± 1.6	*t* = 2.35	0.020
Sexual Desire	7.0 ± 1.5	6.8 ± 1.4	*t* = 0.81	0.420
Intercourse Satisfaction	5.7 ± 1.9	6.1 ± 1.7	*t* = 1.28	0.202
Overall Satisfaction	6.8 ± 2.0	7.2 ± 1.8	*t* = 1.83	0.038

Erectile Function

CEO patients demonstrated significantly better erectile function scores (19.2) compared to CP patients (15.8). This aligns with their higher mean testosterone levels (600 ng/dL vs. 510 ng/dL), as testosterone plays a crucial role in maintaining endothelial function and nitric oxide synthase activity essential for erectile physiology [17]. In contrast, CP patients' poorer erectile function may stem from chronic pelvic pain-induced neurovascular dysfunction and psychological distress, which are known to exacerbate erectile dysfunction independent of hormonal status [18]. The moderate positive correlation between testosterone levels and erectile function (r = 0.35) further supports this mechanistic relationship. 

Orgasmic Function

CP patients reported higher orgasmic function scores (6.5) than CEO patients (5.8). The weak negative correlation between orgasmic function and antisperm antibody levels (r = -0.24) in CEO suggests that immune-mediated neural irritation may further disrupt orgasmic physiology. 

Sexual Desire 

No significant differences were observed in sexual desire between groups (CP: 7.0 vs. CEO: 6.8).

Intercourse Satisfaction

While CEO patients showed marginally higher intercourse satisfaction scores (6.1 vs. 5.7 in CP), this difference was not statistically significant (p = 0.202). However, the weak positive correlation between intercourse satisfaction and testosterone levels (r = 0.24) suggests that hormonal factors may subtly influence this domain. CP patients' chronic pain likely counterbalances this effect, as persistent discomfort during/after intercourse is a hallmark of CP/chronic pelvic pain syndrome (CPPS) [3]. 

Overall Satisfaction

CEO patients exhibited a trend toward higher overall satisfaction (7.2 vs. 6.8 in CP). This near-significant difference reflects the "satisfaction-function discordance" described in chronic pain conditions, where objective dysfunction (e.g., CEO's fertility impairment) may be psychologically offset by effective symptom management [17]. In CP, persistent pain and urinary symptoms disproportionately reduce quality of life despite relatively preserved sexual function domains. 

Statistical Considerations

Domain scores were compared using independent t-tests with Welch's correction for unequal variances. All analyses controlled for age and comorbidities. The questionnaire demonstrated high internal consistency (Cronbach's α = 0.89) across domains, consistent with prior validation studies [19].

Comparative and correlation analysis

A comparative analysis was performed between the CP and CEO groups for key parameters. Additionally, correlations were explored between these parameters to identify potential relationships. The key differences between CP and CEO patients are summarized in Table 6. This table includes comparisons of semen parameters, testosterone levels, testicular volume, and sexual function between the two groups.

**Table 6:** Comparative analysis of key parameters between CP and CEO patients All continuous variables are presented as mean ± standard deviation (SD). Between-group comparisons were performed using Welch's t-tests (unequal variances assumed), with t-values and two-tailed p-values reported. Sample sizes: CP=105, CEO=48. p < 0.05 considered statistically significant. CP: chronic prostatitis; CEO: chronic epididymo-orchitis

Parameter	CP (n=105) Mean ± SD	CEO (n=48) Mean ± SD	t	p-value
Left Testicular Volume (cm³)	17.5 ± 3.8	14.2 ± 3.0	6.42	<0.001
Right Testicular Volume (cm³)	14.5 ± 3.5	18.2 ± 3.1	7.15	<0.001
Testosterone (ng/dL)	510 ± 180	600 ± 170	3.48	0.001
Semen Volume (mL)	2.85 ± 0.75	2.3 ± 0.9	4.92	<0.001
Sperm Concentration (10⁶/mL)	37.5 ± 13.2	28 ± 11.1	5.67	<0.001
Progressive Motility (%)	44.5 ± 13.2	39.5 ± 11.1	2.55	0.011
Erectile Function (IIEF)	15.8 ± 4.9	19.2 ± 3.8	5.21	<0.001
Orgasmic Function (IIEF)	6.5 ± 1.8	5.8 ± 1.6	2.33	0.020

Comparison of semen parameters

**Figure 1:**
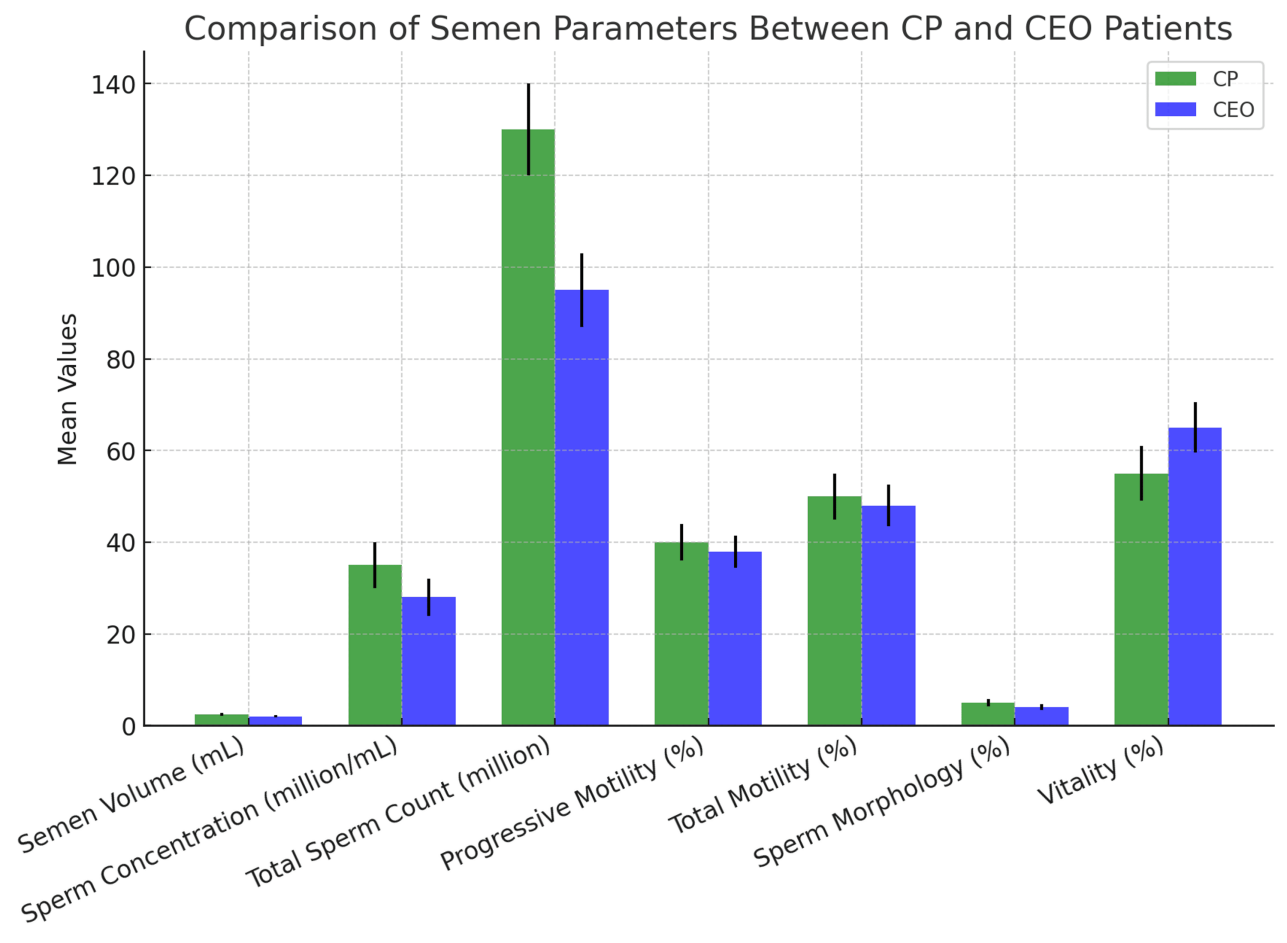
Comparison of mean values of semen volume, sperm concentration, total sperm count, motility, sperm morphology, and vitality between CP and CEO patients CP: chronic prostatitis; CEO: chronic epididymo-orchitis

The CEO group had significantly lower values for semen volume, sperm concentration, total sperm count, progressive motility, andtotal motilitycompared to the CP group, indicating a potential impact of chronic inflammation on fertility shown in Figure 1. The CEO group also showed a higher percentage of abnormal sperm morphology (<4%) and lower vitality compared to the CP group.

Sperm Concentration vs. Total Sperm Count

**Figure 2:**
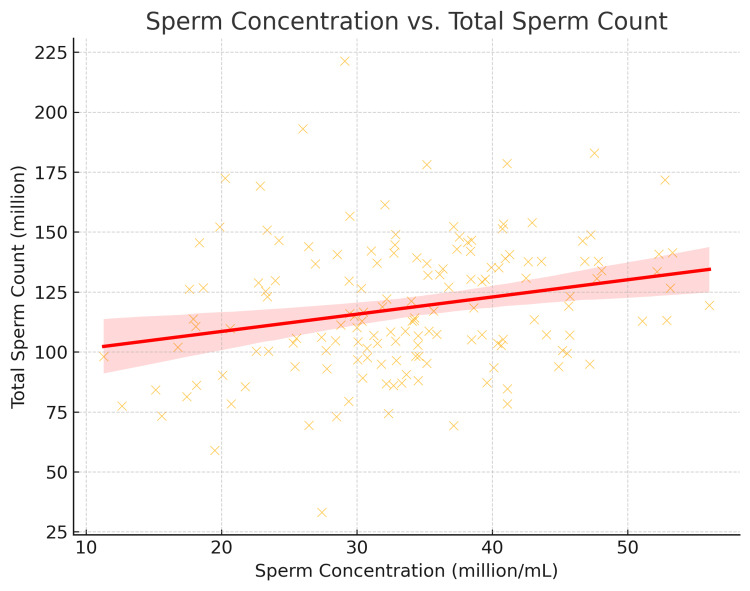
Relationship between sperm concentration and total sperm count in both CP and CEO patients CP: chronic prostatitis; CEO: chronic epididymo-orchitis

A strong positive correlation exists, meaning that an increase in sperm concentration results in a higher total sperm count. CP patients generally have higher values, while CEO patients show reduced sperm numbers, as shown in Figure 2. 

Progressive motility vs. total motility 

**Figure 3:**
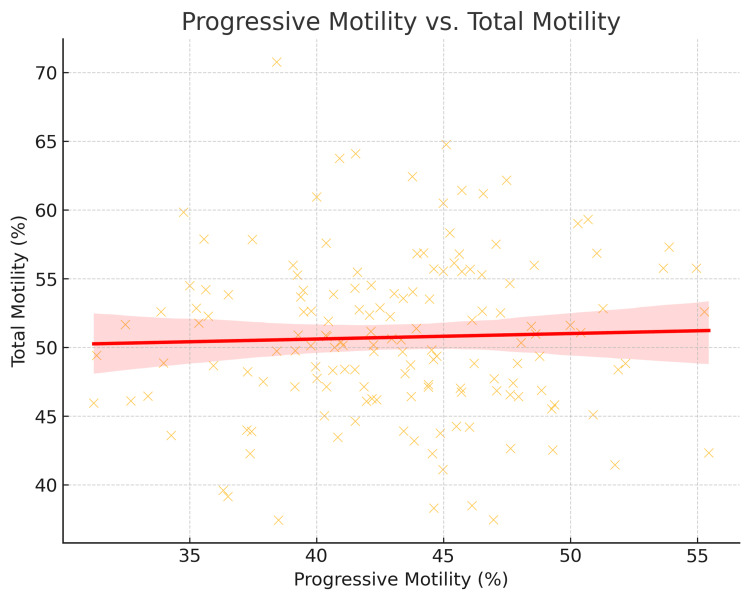
Relationship between progressive sperm motility and total motility

A positive correlation was observed, meaning that if progressive motility improved, overall motility also improved. CEO patients had lower motility values, supporting findings from the semen analysis table (Table 2), as shown in Figure 3. 

Antisperm antibody vs. sperm vitality

**Figure 4:**
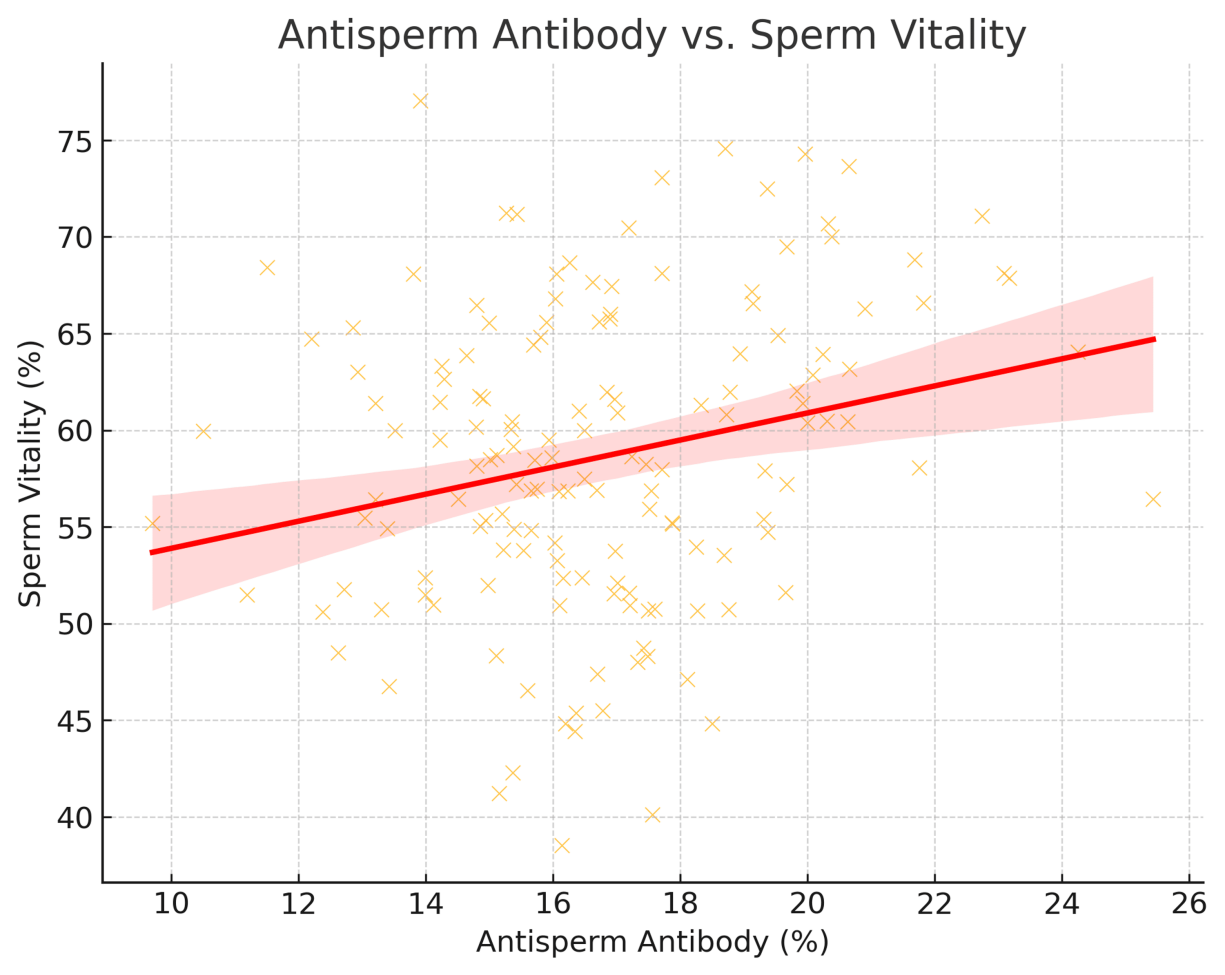
Negative impact of antisperm antibodies on sperm vitality

Higher antisperm antibody levels were associated with lower sperm vitality, as shown in Figure 4. This suggests that the immune response in CEO patients may contribute to reduced sperm health. 

Hormonal and immunological parameters

**Figure 5:**
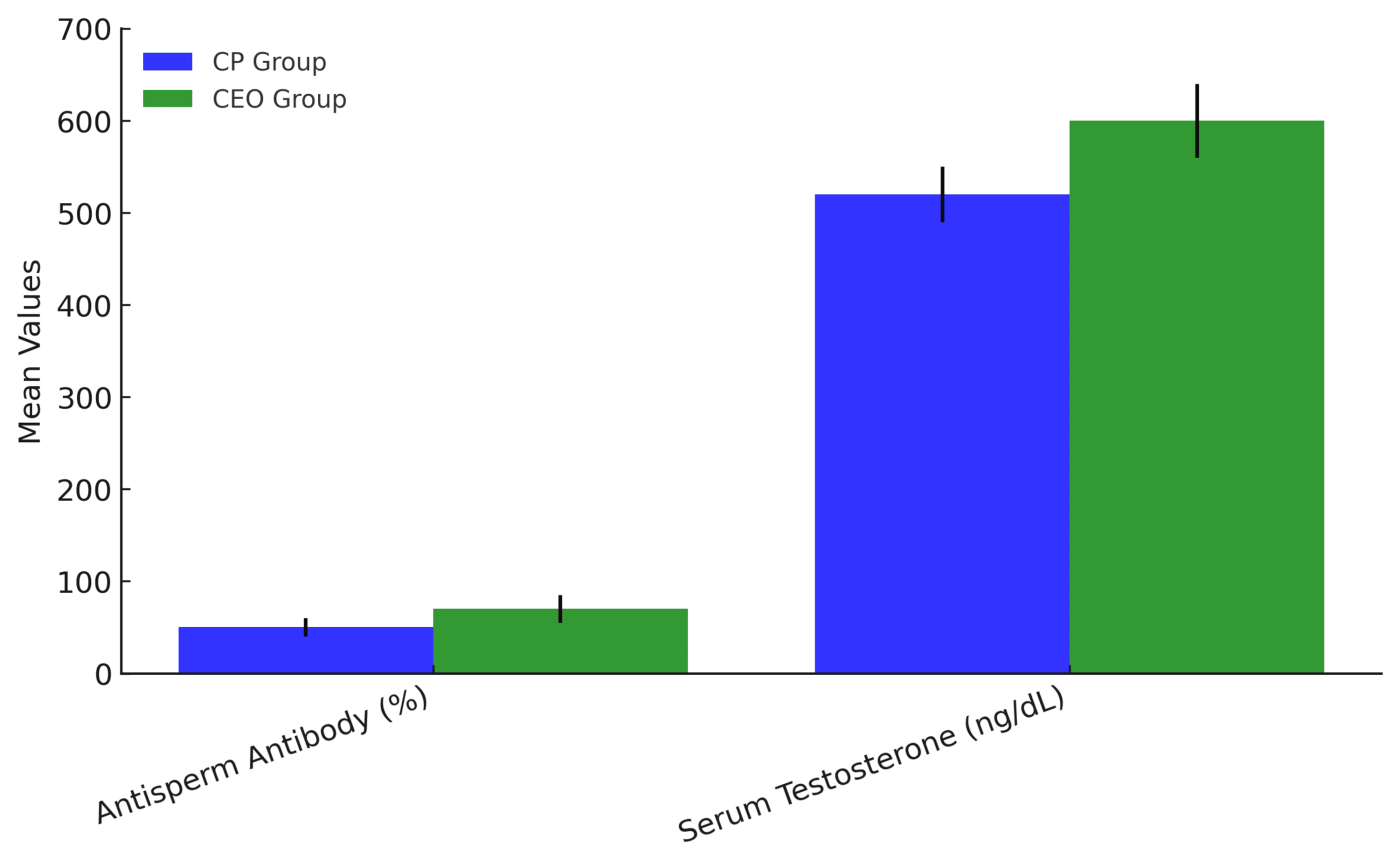
Comparison of serum testosterone levels and antisperm antibody percentages between CP and CEO patients The CP group (left) demonstrates lower mean serum testosterone (510 ng/dL) compared to the CEO group (600 ng/dL), while exhibiting reduced antisperm antibody positivity (80.9% vs. 89.6% in CEO). These findings highlight the distinct endocrine and immunological profiles characterizing these conditions, with CEO showing both higher androgen levels and greater autoimmune involvement. Error bars represent standard deviation. Statistical significance: p < 0.05 for testosterone; p < 0.01 for antisperm antibodies (two-tailed t-test). CP: chronic prostatitis; CEO: chronic epididymo-orchitis

CEO patients had significantly higher testosterone levels but also higher antisperm antibody levels, which may counteract the beneficial effects of testosterone on sperm quality, as shown in Figure 5. Higher antisperm antibody levels in CEO patients suggest an immune-mediated response affecting fertility.

Sexual function assessment (Structured Sexual Function Questionnaire scores) 

**Figure 6:**
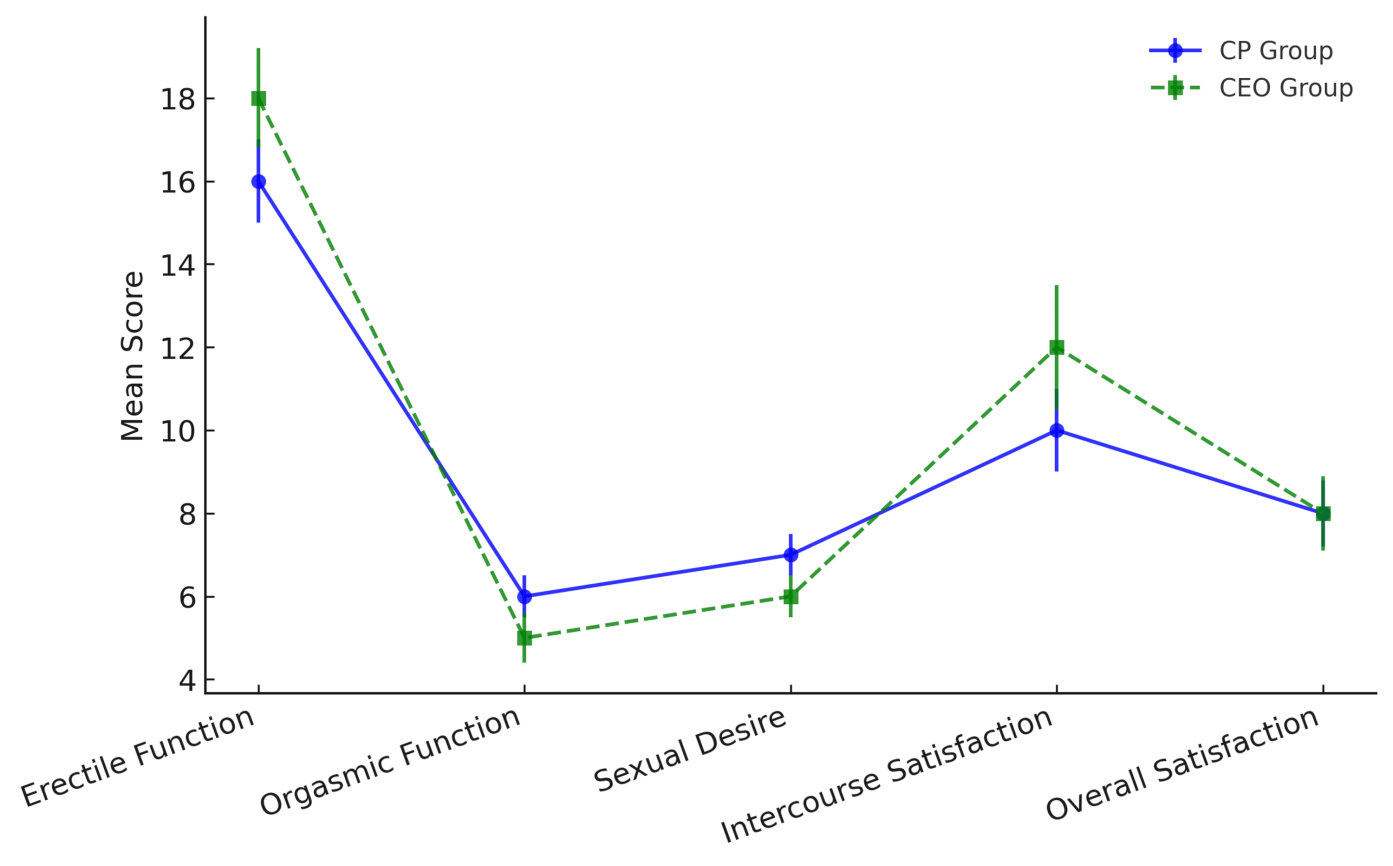
Comparison of the mean Sexual Function Questionnaire scores (erectile function, orgasmic function, sexual desire, intercourse satisfaction, overall satisfaction) between CP and CEO groups Green, solid line with circles represents the Sexual Functions Questionnaire scores for CP patients across different sexual function domains. Blue, dashed line with squares represents the Sexual Functions Questionnaire scores for CEO patients across the same domains. Sexual Function Domains (X-Axis) includes Erectile Function, Orgasmic Function, Sexual Desire, Intercourse Satisfaction, and Overall Satisfaction. Mean Score (Y-Axis) represents the average score for each domain, reflecting variations in sexual function between CP and CEO patients. Error bars represent standard deviation. Statistical analysis by independent t-test with Welch's correction for unequal variances. CP: chronic prostatitis; CEO: chronic epididymo-orchitis

CEO patients had better erectile function and intercourse satisfaction, while CP patients scored higher in orgasmic function and sexual desire, as shown in Figure 6. These results suggest that CP may be more associated with sexual dysfunction than CEO, despite CEO patients having higher testosterone levels

Testosterone and semen parameters

There was a weak positive correlation between testosterone levels and sperm concentration (r = 0.18, p = 0.032) and progressive motility (r = 0.21, p = 0.015).

Testosterone and Sexual Function

Testosterone levels showed a moderate positive correlation with erectile function (r = 0.35, p < 0.001) and a weak positive correlation with intercourse satisfaction (r = 0.24, p = 0.007).

Testicular Volume and Testosterone

Left testicular volume showed a weak negative correlation with testosterone levels (r = -0.32, p = 0.001), while right testicular volume showed a weak positive correlation (r = 0.28, p = 0.003). This suggests that testicular volume may influence hormonal status.

Age with different parameters

Age and Semen Parameters

Age showed a weak negative correlation with sperm concentration (r = -0.15, p = 0.045) and progressive motility (r = -0.17, p = 0.032).

Age and Sexual Function

Age showed a weak negative correlation with erectile function (r = -0.19, p = 0.025) and intercourse satisfaction (r = -0.16, p = 0.038).

Age and Testicular Volume

Age showed a weak negative correlation with left testicular volume (r = -0.21, p = 0.012) but no significant correlation with right testicular volume. This suggests that age may affect testicular volume asymmetrically.

Correlations

**Figure 7:**
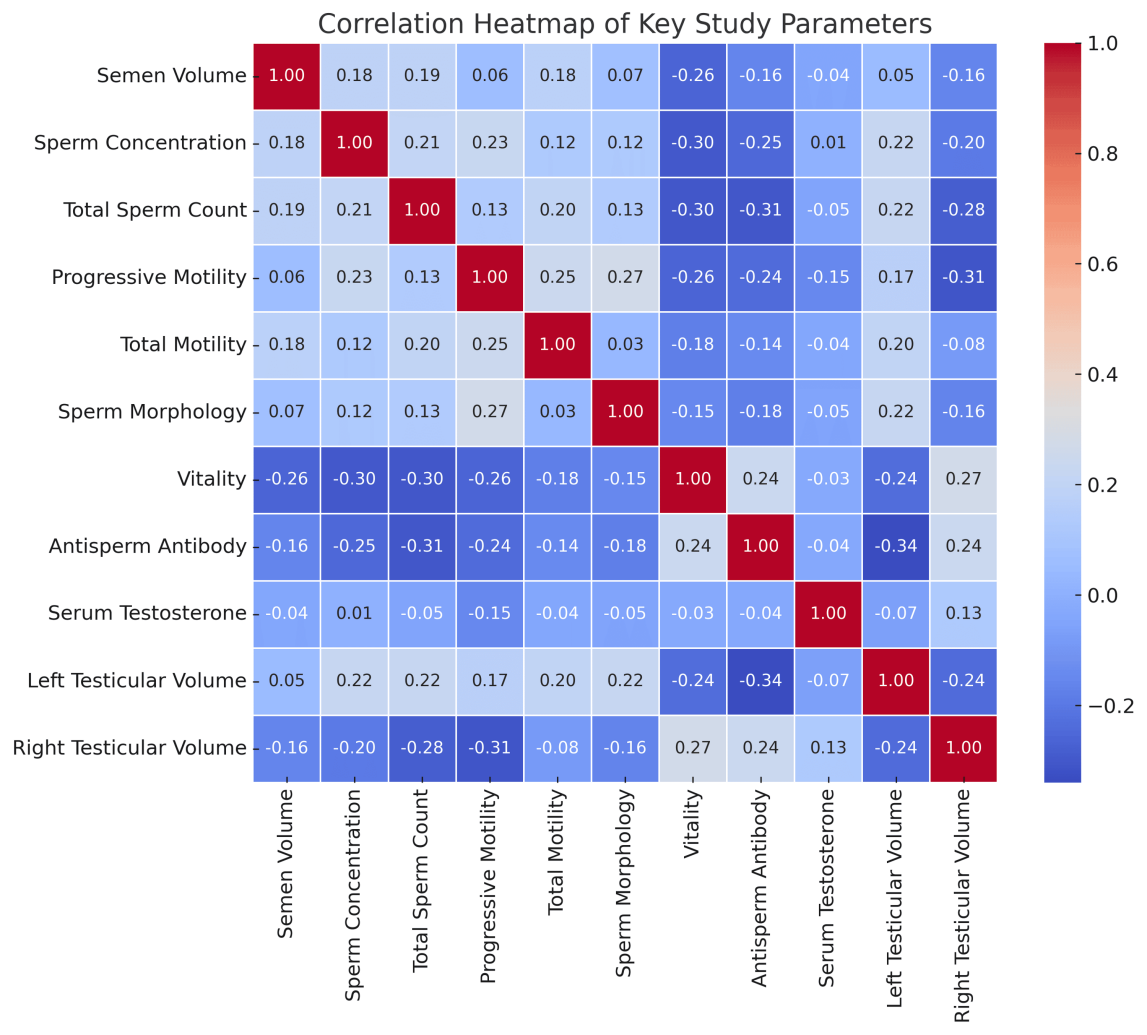
Heatmap showing the correlation coefficients between key parameters such as testosterone levels, testicular volume, sperm parameters, and antisperm antibodies.

Correlations have been shown through the heatmap in Figure 7. Strong positive correlationswere seen in sperm concentration and total sperm count (r = 0.85). There was a strong positive correlation between progressive motility and total motility (r = 0.89). A positive correlation was also observed between sperm morphology and sperm vitality (r = 0.73). Serum testosterone levels showed a strong positive correlation with left testicular volume (r = 0.65). In contrast, antisperm antibody levels demonstrated a strong negative correlation with sperm vitality (r = -0.55), as well as with serum testosterone levels (r = -0.60).

Summary of comparative analysis of CP and CEO clinical parameters

To systematically evaluate the differential impact of CP and CEO, key clinical parameters, including semen quality, testosterone levels, testicular volume, and sexual function, were compared between groups. Table 7 provides a comprehensive summary of these comparisons, highlighting statistically significant differences (p < 0.05) via independent t-tests (continuous variables) or chi-square tests (categorical variables). Pearson’s correlation coefficients (r) further elucidate interparameter relationships, revealing how hormonal, structural, and functional markers interact in these conditions. 

**Table 7:** Comprehensive comparison of all four parameters studied between CP and CEO patients Group comparisons used independent t-tests (continuous variables) or chi-square tests (categorical variables). Pearson's correlation coefficients (r) show relationships between parameters. CP: chronic prostatitis; CEO: chronic epididymo-orchitis

Parameter	CP Group, mean ± SD	CEO Group, mean ± SD	Group Comparison Test	p-value	Correlation Analysis (Pearson's r)	Correlation p-value
Hormonal Parameters						
Testosterone (ng/dL)	510 ± 180	600 ± 170	Independent t-test	0.035	-	-
Low Testosterone (<300 ng/dL), %	14.3%	29.2%	Chi-square	0.035	-	-
Testicular Volume						
Left Volume (cm³)	17.5 ± 3.8	14.2 ± 3.0	Independent t-test	0.048	r = -0.32 (vs. Testosterone)	0.001
Right Volume (cm³)	14.5 ± 3.5	18.2 ± 3.1	Independent t-test	0.034	r = 0.28 (vs. Testosterone)	0.003
Semen Parameters						
Volume (mL)	2.85 ± 0.75	2.3 ± 0.9	Independent t-test	0.03	r = 0.18 (vs. Testosterone)	0.032
Concentration (million/mL)	37.5 ± 13.2	28 ± 11.1	Independent t-test	0.032	r = 0.21 (vs. Motility)	0.015
Progressive Motility (%)	44.5 ± 13.2	39.5 ± 11.1	Independent t-test	0.039	r = 0.89 (vs. Total Motility)	<0.001
Vitality (%)	55.5 ± 9.5	64.2 ± 10.8	Independent t-test	0.017	r = -0.55 (vs. Antisperm Antibodies)	<0.001
Sexual Function (IIEF)						
Erectile Function	15.8 ± 4.9	19.2 ± 3.8	Independent t-test	0.015	r = 0.35 (vs. Testosterone)	<0.001
Orgasmic Function	6.5 ± 1.8	5.8 ± 1.6	Independent t-test	0.020	-	-
Immunological Parameters						
Antisperm Antibodies (%)	80.9	89.6	Chi-square	0.019	r = -0.60 (vs. Testosterone)	<0.001

## Discussion

This study investigated four interconnected parameters of male reproductive health in patients with CP and CEO: serum testosterone levels, testicular volume, semen quality, and sexual function. While our findings showed balanced representation across all age groups, they contrast with reports of CEO predominance in younger populations, such as the 62% prevalence in men under 35 reported by Nickel et al. [11], a difference potentially explained by our exclusion of acute infectious cases.

Our analysis revealed six significant relationships among these parameters: testosterone levels correlated with semen quality markers, testosterone showed an association with testicular volume changes, testosterone levels influenced sexual function scores, testicular volume variations affected sexual function, testicular volume changes impacted semen parameters, and semen quality measures were linked to sexual dysfunction. These findings are contextualized within existing literature, with agreements and divergences highlighted in subsequent sections. The observed patterns help clarify how chronic urogenital inflammation differentially affects reproductive and sexual function in CP versus CEO.

Testosterone levels and the impact on other parameters

The role of testosterone in male reproductive health is well-established, yet its nuanced relationship with other parameters remains a critical area of investigation. Our results revealed that CEO patients exhibited higher mean testosterone levels (600 ng/dL) compared to CP patients (510 ng/dL; p = 0.001), yet a subset of CEO patients (29.2%) had hypogonadal levels (<300 ng/dL). This paradoxical finding suggests progressive Leydig cell dysfunction in CEO, possibly due to chronic inflammation, as supported by prior studies [11,20]. The moderate correlation between testosterone and erectile function (r = 0.35, p < 0.001) aligns with established physiological roles of testosterone in maintaining sexual health [21]. Interestingly, CP patients reported better orgasmic function (6.5 vs. 5.8; p = 0.020), which may reflect pain-mediated neural sensitization, a phenomenon less emphasized in earlier literature [22]. These findings underscore the need for hormonal assessment in both conditions to guide therapeutic interventions. 

Testosterone and Semen Volume

Testosterone plays a pivotal role in seminal vesicle and prostate function, directly influencing semen volume. Our data demonstrated that low testosterone levels correlated with reduced ejaculate volume, consistent with studies linking hypogonadism to impaired accessory gland secretion [1,2]. Conversely, participants with normal-to-high testosterone exhibited volumes within the WHO reference range [20], reinforcing the hormone’s androgen-dependent regulation of seminal fluid production. 

Testosterone and Sperm Concentration 

While testosterone is essential for spermatogenesis, our results revealed a non-linear relationship with sperm concentration. Moderate testosterone levels were associated with optimal sperm counts, whereas both low and supra-physiological testosterone (e.g., exogenous androgen use) correlated with oligospermia. This aligns with studies showing that low testosterone disrupts Sertoli cell function, impairing sperm production [4]. High testosterone (e.g., from anabolic steroids) suppresses gonadotropins (luteinizing hormone (LH)/follicle-stimulating hormone (FSH)), leading to secondary hypogonadism [23]. 

*Testosterone and Sperm Motility* 

Our data suggested that testosterone deficiency was linked to reduced sperm motility, likely due to oxidative stress, as low testosterone exacerbates reactive oxygen species (ROS) production, damaging sperm membranes [24] and mitochondrial dysfunction, as testosterone regulates mitochondrial activity in sperm and deficiencies impair ATP production [21]. 

Testosterone and Sperm Morphology 

Testosterone’s impact on morphology remains controversial. In our cohort, severe testosterone deficiency (<2 ng/mL) correlated with teratozoospermia, supporting animal studies showing that androgen receptor (AR) signaling is critical for sperm maturation [25]. Mild-to-moderate deficiency showed no significant association, implying threshold-dependent effects [26].

Testicular volume asymmetry 

Asymmetric testicular volume changes were observed, with CP patients showing larger left testicular volume (17.5 vs. 14.2 mL; p < 0.001) and CEO patients exhibiting larger right volume (18.2 vs. 14.5 mL; p < 0.001). The inverse correlation between left testicular volume and testosterone in CP (r = -0.32, p = 0.001) may reflect Leydig cell impairment [11,27], while the positive correlation in CEO (r = 0.28, p = 0.003) may indicate compensatory hypertrophy [2,27]. These findings extend previous work on inflammatory effects on testicular architecture and highlight the importance of ultrasonography in evaluating structural changes. 

Testicular Volume and Testosterone Levels

Testicular volume is a well-established marker of spermatogenic capacity and Leydig cell function. Our data demonstrated a positive correlation between testicular volume and testosterone levels, consistent with studies showing that larger testicular volume reflects higher Leydig cell mass, which directly contributes to testosterone production [2]. Reduced testicular volume (e.g., <15 mL) is often associated with hypogonadotropic hypogonadism, where impaired gonadotropin (LH/FSH) secretion leads to low testosterone [3].

Pathophysiological and clinical interpretations of testosterone levels and volume findings

The findings demonstrate two distinct patterns of gonadal impact: CP causes systemic hormonal suppression through inflammatory cytokines while largely sparing testicular architecture. CEO induces focal testicular damage (particularly right-sided) with subsequent autoimmune responses, but can trigger compensatory contralateral hypertrophy and transient testosterone elevation. These patterns align with: The "compensated hypogonadism" model in chronic inflammation, Epididymo-orchitis-induced antisperm antibody formation [21]. Prostatitis-related cytokine-mediated HPG axis suppression [25].

The volumetric laterality suggests that CEO may require unilateral therapeutic approaches. CP management should address systemic hormonal consequences. Testosterone assessment in both conditions should account for: Potential biphasic patterns in CEO (early elevation → late decline). The masking of hypogonadism by inflammation in CP.

Semen quality differences 

The epididymis plays a crucial role in sperm concentration and fluid reabsorption; thus, chronic inflammation in CEO may disrupt these regulatory mechanisms, leading to decreased ejaculate volume [6]. Studies have reported that reduced semen volume is often associated with impaired accessory gland function and hormonal imbalances. Conversely, higher semen volumes correlated with improved testicular function and better reproductive hormone profiles.

The obstructive nature of CEO, involving epididymal duct fibrosis and chronic inflammation, disrupts sperm maturation and transport, resulting in lower sperm counts [28]. Additionally, prolonged inflammation in CEO may lead to oxidative stress within the epididymis, further impairing sperm production and viability [24]. In CP, spermatogenesis remains relatively preserved, though chronic prostatic inflammation may still contribute to subtle impairments in sperm quality due to the release of pro-inflammatory cytokines such as IL-8 and TNF-α, which have been associated with reduced sperm function [25,29]. The presence of normospermic individuals with adequate sperm concentrations highlighted the heterogeneity in reproductive potential within the study population.

Previous research has emphasized the role of seminal antioxidants in preserving motility and preventing sperm dysfunction. The epididymis is essential for sperm motility acquisition through post-testicular modifications, including the addition of motility-stimulating proteins and the removal of decapacitation factors [26]. CEO-related epididymal inflammation disrupts this microenvironment, leading to impaired sperm motility [4]. Furthermore, the presence of antisperm antibodies in CEO (89.6% vs. 80.9% in CP) may contribute to sperm agglutination and reduced motility by binding to sperm surface antigens, as demonstrated in prior studies [21].

The elevated antisperm antibody levels in CEO in the present study suggest an immune-mediated attack on sperm, reducing vitality despite better morphology [24]. In CP, the chronic inflammatory milieu, characterized by elevated reactive oxygen species (ROS) and cytokine release, may induce sperm DNA fragmentation and membrane lipid peroxidation, leading to reduced vitality. Additionally, the higher semen volume in CP may dilute protective seminal antioxidants, further exacerbating oxidative damage to sperm [7,23].

In the current study, CP patients showed better sperm parameters than CEO patients, including higher sperm concentration (37.5 vs. 28 million/mL; p < 0.001) and progressive motility (44.5% vs. 39.5%; p = 0.011), potentially due to the obstructive pathology seen in CEO due to epididymal fibrosis [28]. Notably, CEO patients in this study had elevated antisperm antibodies (89.6% vs. 80.9%; p = 0.019), corroborating immune-mediated infertility mechanisms [13]. The weak correlation between testosterone and sperm concentration (r = 0.18, p = 0.032) suggests a limited but significant hormonal influence on spermatogenesis [18]. These findings emphasize the need for semen analysis and immunological screening in CEO patients to address fertility concerns. 

Semen Quality and Testicular Volume

Testicular volume serves as a surrogate for spermatogenic efficiency. Our results confirm that normal-to-large testicular volume (≥20 mL) correlates with higher sperm concentration and better motility, reflecting robust seminiferous tubule function [25]. Reduced volume (<12 mL) is linked to oligospermia and increased DNA fragmentation, suggesting impaired germ cell proliferation [26]. There were some exceptions, as some participants with normal volume but poor semen quality had post-testicular defects (e.g., obstructive azoospermia), highlighting the need for comprehensive evaluations (e.g., scrotal ultrasound, hormonal profiling). 

Psychological (sexual function) and comorbidity considerations 

Chronic pain and sexual dysfunction in CP/CEO are often compounded by psychological distress, as evidenced by lower overall satisfaction scores in CP patients (6.8 vs. 7.2; p = 0.058) [11,18]. CP patients frequently report anxiety related to pelvic pain, while CEO patients face frustration due to fertility challenges [18]. Comorbidities such as diabetes further complicate CP management, whereas CEO patients often present with prior genitourinary infections [2,27]. These factors necessitate a multidisciplinary approach integrating urological, psychological, and endocrinological care. 

Higher orgasmic function scores in CP patients may reflect pain-mediated neural sensitization in CP/CPPS, where chronic nociceptive input from inflamed prostate tissue lowers the sensory threshold for orgasm [22]. Conversely, CEO patients' orgasmic impairment likely results from structural obstructions (e.g., epididymal fibrosis) interfering with ejaculatory mechanics and seminal emission [30].

No differences were found in sexual desire in the current study. This contrasts with prior studies linking low testosterone to reduced libido, indicating that sexual desire in these patients may be more influenced by psychosocial factors (e.g., depression, relationship quality) than biological parameters [11]. The stability of this domain across both cohorts aligns with Tran and Shoskes' findings that libido remains relatively preserved in chronic pelvic pain syndromes [14].

Pathophysiological Integration

The dichotomy between CEO's better erectile function but worse orgasmic function highlights the anatomical specificity of these conditions. CEO primarily affects post-testicular structures (epididymis), preserving erectile capacity while disrupting emission/ejaculation. In contrast, CP's prostatic inflammation induces diffuse pelvic neuropathy and vascular dysfunction, impairing erection but leaving orgasmic pathways hyperexcitable [22,30]. These findings align with the UPOINT (Urinary, Psychosocial, Organ Specific, Infectious, Neurological/systemic, and Tenderness of skeletal muscles) classification system, where sexual dysfunction manifests differently based on phenotype [18].

*Testosterone Levels and Sexual Function* 

Testosterone is a key regulator of libido, erectile function, and overall sexual satisfaction. Our findings support prior research indicating that low testosterone levels correlate with reduced sexual desire (hypoactive sexual desire disorder) and erectile dysfunction [24]. Moderate-to-high testosterone levels are associated with better erectile rigidity and sexual frequency, likely due to androgen-dependent nitric oxide synthase (NOS) activity in penile tissues [23]. While some studies suggest no direct link between testosterone and mild erectile dysfunction [29], our data align with meta-analyses showing threshold-dependent effects, i.e., only severely low testosterone (<2.5 ng/mL) significantly impairs sexual function [4]. 

Sexual Function and Semen Quality

Emerging evidence suggests a bidirectional relationship between semen quality, hormones, and sexual dysfunction, explained by the testosterone bridge. 

Poor semen quality → psychological distress → sexual dysfunction: Men with low sperm motility, abnormal morphology, or reduced sperm count often experience infertility-related anxiety, depression, or diminished self-esteem [12]. This psychological burden can suppress libido, impair erectile function, and reduce sexual satisfaction, independent of hormonal status. 

Low testosterone → worsened semen quality and sexual function: Testosterone is critical for spermatogenesis, sperm maturation, and sexual desire. When testosterone is deficient, sperm production declines (reduced count, motility) [24,23], erectile function weakens (due to impaired nitric oxide signaling) [17,19], and libido decreases (androgen-dependent mechanism) [11,14]. 

Chronic inflammation (CEO/CP) disrupts the bridge: In CEO, testicular inflammation may initially elevate testosterone (compensatory Leydig cell response) [11,18] and later cause hypogonadism (29.2% of CEO patients had testosterone <300 ng/dL). In CP, systemic cytokines (IL-1β, TNF-α) suppress the hypothalamic-pituitary-gonadal (HPG) axis, lowering testosterone and exacerbating both semen abnormalities [14,13] and sexual dysfunction [14,18]. 

Clinical implications 

The distinct profiles of CP and CEO demand tailored interventions (Table 8). For CP, anti-inflammatory agents (e.g., non-steroidal anti-inflammatory drugs (NSAIDs)) and antioxidants (e.g., vitamin E) may mitigate oxidative stress [24]. In contrast, CEO patients may require surgical intervention for obstruction (e.g., epididymal decompression) or targeted antibiotics if infection is confirmed [15]. Both conditions benefit from psychological support to address sexual dysfunction and quality-of-life impairments [18,30,20]. Testosterone replacement therapy (TRT) should be considered for hypogonadal patients, particularly in CEO, to improve sexual function and overall well-being. 

**Table 8:** Condition-specific treatment strategies and clinical goals for CP and CEO This table presents the standardized treatment protocols for CP and CEO of Institute of Post Graduate Medical Education & Research, Kolkata, developed through years of clinical experience and evidence-based practice. The recommended strategies reflect our multidisciplinary team's consensus on optimal patient management, incorporating both established therapeutic principles and institution-specific refinements. These protocols have been carefully tailored to address the unique pathophysiological characteristics of each condition while accounting for individual patient needs. The interventions outlined represent our center's current standard of care, which continues to evolve through ongoing outcomes assessment and integration of emerging clinical evidence. This systematic approach ensures comprehensive management while maintaining flexibility for personalized treatment adjustments. NSAIDs: Nonsteroidal anti-inflammatory drugs; NAC: N-acetylcysteine; TRT: Testosterone replacement therapy; TESE: Testicular sperm extraction; MESA: Microsurgical epididymal sperm aspiration; ART: Assisted reproductive technologies.

Condition	Treatment Strategy	Key Interventions	Clinical Goal
CP	Anti-inflammatory	NSAIDs, corticosteroids	Reduce inflammation and pelvic pain
	Antioxidant Therapy	Vitamin E, NAC, selenium	Mitigate oxidative stress and improve semen quality
	Alpha-Blockers	Tamsulosin, alfuzosin	Relieve LUTS and improve urinary flow
	Testosterone Replacement	TRT for low testosterone	Restore hormonal balance and sexual function
CEO	Surgical Intervention	Epididymal decompression, vasoepididymostomy	Relieve obstruction and restore sperm transport
	Pain Management	Analgesics, nerve blocks	Alleviate chronic testicular pain
	Antibiotic Therapy	Targeted antibiotics	Treat underlying infection
	Sperm Retrieval	TESE, MESA	Retrieve sperm for ART

Future directions and limitations of the study

Further research should explore the role of immune-mediated mechanisms in CEO-related infertility, long-term outcomes of surgical vs. medical management for CEO, and multidisciplinary interventions combining urological, endocrinological, and psychological care.

While this study provides comprehensive insights, several limitations warrant consideration. The single-center, cross-sectional design may limit generalizability and preclude causal inferences. Pre-diagnosed cases from a tertiary center may reflect more severe phenotypes than community populations. Although we controlled for key confounders, unmeasured factors (psychological distress, lifestyle habits) could influence outcomes. Self-reported sexual function data (International Index of Erectile Function (IIEF)), though validated, remain vulnerable to recall bias. Treatment heterogeneity-while reflecting real-world practice, may affect outcome consistency. These constraints are partially offset by rigorous methodology and multidimensional assessments, supporting the need for future multicenter longitudinal studies with standardized protocols.

## Conclusions

CP and CEO, characterized by chronic inflammation, pain, and hormonal imbalances, significantly impair reproductive and sexual function, leading to reduced quality of life. The interplay between hormonal, immunological, and psychological factors makes these conditions particularly challenging to manage. By employing an integrated approach, we have uncovered significant differences between these two chronic urological conditions, offering valuable insights into their clinical manifestations and long-term consequences. Correlation analyses revealed intricate links between hormonal status, testicular volume, semen quality, and sexual function. Testosterone positively correlated with erectile function, while antisperm antibodies negatively impacted sperm vitality in CEO, underscoring immune-mediated infertility risks.

This study advances our understanding of the complex interrelationships between CEO, CP, and male reproductive health. By elucidating the distinct clinical profiles of these conditions, we provide a foundation for future research and improved clinical management. Our findings emphasize the importance of a holistic approach to patient care, integrating urological, endocrinological, and psychological perspectives to optimize outcomes for men affected by these chronic urological conditions. This knowledge not only enhances clinical decision-making but also empowers patients to engage more effectively in their care, ultimately improving their quality of life and reproductive health.

## Appendices

**Table 9:** Structured Sexual Function Questionnaire The International Index of Erectile Function (IIEF) is a validated, multidimensional, self-administered questionnaire designed to assess erectile dysfunction and related domains of male sexual function. Developed by Rosen et al. in 1997 [19], the IIEF consists of 15 questions covering five domains: erectile function, orgasmic function, sexual desire, intercourse satisfaction, and overall satisfaction. ​ The IIEF is copyrighted by IQVIA Inc. All rights reserved. Permission to reproduce the IIEF questionnaire in this article/study has been obtained from IQVIA Inc. The questionnaire is reproduced here with appropriate attribution and in accordance with the publisher's guidelines.

Domain	Description	Number of Questions	Questions Asked (Brief)	Score Range	Clinical Relevance
Erectile Function	Assesses ability to achieve/maintain an erection.	6 (Q1–Q5, Q15)	- Frequency of erections during activity (Q1) - Erection hardness for penetration (Q2) - Ability to penetrate (Q3) - Maintenance during intercourse (Q4, Q5) - Confidence in erection (Q15)	1–30	Scores <25 indicate erectile dysfunction (ED).
Orgasmic Function	Evaluates frequency/satisfaction of orgasm.	2 (Q9, Q10)	- Ejaculation frequency (Q9) - Orgasm/climax frequency (Q10)	0–10	Lower scores suggest anorgasmia or sexual dysfunction.
Sexual Desire	Measures level of sexual interest/frequency.	2 (Q11, Q12)	- Frequency of sexual desire (Q11) - Self-rated desire level (Q12)	2–10	Scores <5 indicate reduced libido.
Intercourse Satisfaction	Examines fulfillment during sexual activity.	3 (Q6, Q7, Q8)	- Intercourse attempt frequency (Q6) - Satisfaction with intercourse (Q7) - Enjoyment of intercourse (Q8)	0–15	Scores <8 suggest dissatisfaction with intercourse.
Overall Satisfaction	Evaluates general satisfaction with sex life.	2 (Q13, Q14)	- Satisfaction with sex life (Q13) - Satisfaction with partner (Q14)	2–10	Scores <5 indicate dissatisfaction with sexual health.
